# Impact of Modified Lactoperoxidase Systems on Glycolytic Metabolism and Virulence Factors in *Streptococcus mutans*

**DOI:** 10.3390/ijms27020799

**Published:** 2026-01-13

**Authors:** Marcin Rafał Magacz, Anna Skalniak, Paweł Mamica, Wiktoria Pepasińska, Anna Maria Osyczka, Grzegorz Tylko, Wirginia Krzyściak

**Affiliations:** 1 Department of Medical Diagnostics, Faculty of Pharmacy, Jagiellonian University Medical College, Medyczna 9, 30-688 Kraków, Poland; pawmamic@gmail.com (P.M.); wiktoria.pepasinska@gmail.com (W.P.); 2 Center for the Development of Therapies of Civilization and Age-Related Diseases, Jagiellonian University Medical College, Skawińska 8, 31-066 Kraków, Poland; 3 Department of Cell Biology and Imaging, Institute of Zoology and Biomedical Research, Jagiellonian University, Gronostajowa 9, 30-387 Kraków, Poland

**Keywords:** lactoperoxidase system, *Streptococcus mutans*, biofilm, pyruvate metabolism, virulence genes, thiocyanate, iodide, selenocyanate, dental caries

## Abstract

The lactoperoxidase system (LpoS) is an enzymatic antimicrobial mechanism of saliva that oxidizes (pseudo)halide substrates to reactive compounds capable of limiting microbial growth. This study evaluated how different LpoS variants—utilizing iodide (LpoS-I^−^), thiocyanate (LpoS-SCN^−^), selenocyanate (LpoS-SeCN^−^), and a thiocyanate–iodide mixture (LpoS-SCN^−^ + I^−^)—affect virulence, metabolism, and biofilm structure in *Streptococcus mutans*. Using qRT-PCR, pyruvate assays, MTT reduction, and confocal microscopy, we found that LpoS-I^−^ most effectively reduced *atpD* and ldh expression, impaired acid tolerance, and decreased lactate and pyruvate production. LpoS-SCN^−^ and LpoS-SeCN^−^ also downregulated *atpD* and gtfB, although LpoS-SeCN^−^ upregulated ldh. Despite minimal structural biofilm disruption, LpoS-I^−^ markedly inhibited intracellular and extracellular pyruvate accumulation, suggesting altered glycolytic flux. These findings indicate that iodide-based LPO systems modulate key metabolic and regulatory pathways in *S. mutans* and may hold potential for inclusion in anticaries oral formulations.

## 1. Introduction

The lactoperoxidase enzymatic system (LpoS) is a mucosal defense mechanism, the antimicrobial activity of which is based on the catalyzed oxidation of (pseudo)halide substrates [1,2]. In the oral cavity, this enzyme is secreted by epithelial cells of the submandibular and parotid salivary glands [3]. The physiological concentration of LPO in saliva ranges from 30 to 70 nmol/L, exhibiting diurnal variation with a maximum at midday and dependence on age and hormonal status [2,4].

Under physiological conditions, the main substrate for LPO in the oral cavity is the thiocyanate ion, present in saliva at concentrations of 0.3–3 mM, derived mainly from a diet rich in vegetables [5]. Hydrogen peroxide, essential for the enzymatic reaction, is produced both by host cells (DUOX1/DUOX2 oxidases) and by commensal oral bacteria, particularly streptococci [3,6]. In the (pseudo)halogenation cycle, LPO catalyzes the oxidation of SCN^−^ to hypothiocyanite ions (OSCN^−^) and hypothiocyanous acid (HOSCN), which exhibit selective antimicrobial activity through oxidation of bacterial protein thiol groups [1,7,8,9].

In addition to its physiological thiocyanate substrate, LPO can utilize alternative (pseudo)halide substrates such as iodide (I^−^) and selenocyanate (SeCN^−^) ions [1,10]. The oxidation products of these substrates differ in their antimicrobial spectrum and biocidal potential. Iodide ions are oxidized to various products depending on physicochemical conditions—collectively referred to as reactive iodine species—which exhibit broad-spectrum antimicrobial activity [11], whereas selenocyanate ions form hyposelenocyanous acid (HOSeCN), the properties of which resemble those of thiocyanate-derived products [12]. The LPO system plays a crucial role in maintaining oral microbial homeostasis by regulating the composition of the microbiome and inhibiting the growth of pathogens responsible for oral diseases, including dental caries [2,13,14].

*Streptococcus mutans*, the main etiological agent of dental caries, is characterized by its ability to form biofilms, produce organic acids, and tolerate acidic environments [15,16,17]. This bacterium possesses defense mechanisms against the products of the LPO system, including NADH/hypothiocyanite reductase, which neutralizes OSCN^−^ [18]. Modulation of the LPO system through the use of alternative substrates may represent a strategy to enhance its effectiveness against cariogenic pathogens [19].

Previous studies on the modification of the LPO system have focused primarily on enzymatic analyses and characterization of the antimicrobial properties of individual oxidation products [20]. However, comprehensive studies combining the analysis of the molecular mechanisms of action of modified LPO systems with the assessment of their effects on key virulence factors of *S. mutans*—such as organic acid synthesis, extracellular biofilm matrix formation, and environmental stress tolerance—are lacking.

The aim of this study was to evaluate the effects of modified lactoperoxidase systems utilizing alternative (pseudo)halide substrates (iodide, selenocyanate, and a thiocyanate–iodide mixture) on key pathogenicity mechanisms of *S. mutans*, with particular emphasis on virulence gene expression (*atpD*, *gtfB*, *ldh*), pyruvate metabolism, metabolic activity, and the structure and viability of biofilms.

## 2. Results

### 2.1. Effect of LpoS Systems on the Expression of Virulence Genes

**Figure 1:**
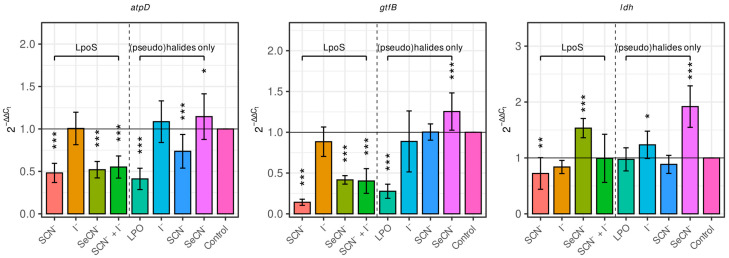
The relative levels of expression of the analyzed virulence genes in the biofilms treated with the tested systems. The expression is presented in relation to the control sample (biofilm with PBS) using the reference gene *gyrA*. The vertical dotted line separates the test samples (LpoSs) from the individual control samples (blank, substrate controls). Data are presented as mean ± SD. * *p* < 0.05, ** *p* <0.01, *** *p* < 0.001 (Dunnett’s test).

Figure 1 shows the relative expression levels of the *atpD*, *gtfB*, and *ldh* genes after treatment with four LpoS LPO-S variants. The thiocyanate (LpoS-SCN^−^), selenocyanate (LpoS-SeCN^−^), and thiocyanate–iodide (LpoS-SCN^−^ + I^−^) systems significantly downregulated *atpD* and *gtfB* expression compared with the control (*p* < 0.05). In contrast, the iodide-based system (LpoS-I^−^) had no significant effect on these genes. A reduction in *ldh* transcript levels was observed following LpoS-SCN^−^ and LpoS-I^−^ treatment, whereas LpoS-SeCN^−^ caused a significant upregulation of *ldh* expression.

Biofilms exposed to (pseudo)halide substrates alone also exhibited altered gene expression: SCN^−^ decreased *atpD* and *ldh* levels, while SeCN^−^ enhanced *gtfB* and *ldh* expression. These findings indicate that both the enzymatic and substrate components modulate the transcriptional response of *S. mutans* biofilms.

### 2.2. The Effect of the Tested Systems on the Production and Secretion of Pyruvate

**Figure 2:**
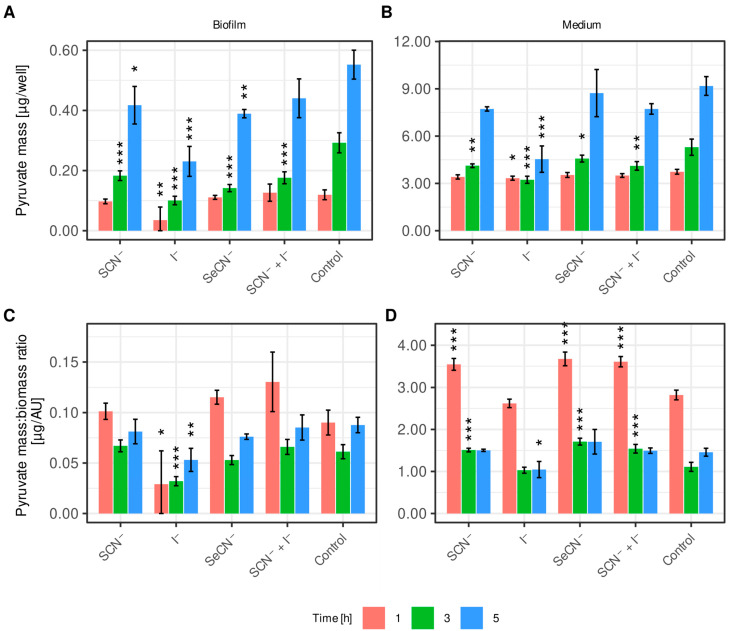
Pyruvate mass (**A**,**B**) and pyruvate/biomass ratio (**C**,**D**) in a well of a 24-well plate/in biofilm at the bottom of the well after treatment with each LpoS variant. X-axis labels indicate the (pseudo)halide substrate used in each LpoS modification. Data are presented as mean ± SD. * *p* < 0.05, ** *p* < 0.01, *** *p* < 0.001 (Dunnett’s test).

Figure 2 illustrates the impact of each LpoS on pyruvate production and its secretion into the medium. Sections A and B depict the mass of pyruvate produced inside *S. mutans* cells (A) and the mass of pyruvate expelled into the medium (B). Sections C and D present equivalent data, displayed as the ratio of the intracellular pyruvate mass (C) or pyruvate mass in medium (D) to total biofilm biomass, as assessed using the CV method. Analyzing the results in this manner normalizes the pyruvate mass in relation to biofilm biomass, as biofilm pyruvate production is directly proportional to its biomass. This approach prevents false conclusions being drawn about *S. mutans* pyruvate metabolism caused by the effect of LpoSs on increasing biofilm biomass. This effect can be observed in Figure 2 as disappearance of statistical significance of the differences in each sample after the mass of produced pyruvate was recalculated in relation to total biomass. Samples that are significantly different from the control in graphs C and D should be considered as those in which the given systems cause a biological effect.

After one hour of exposure, only the iodide-based system (LpoS-I^−^) caused a statistically significant reduction in both intracellular and extracellular pyruvate compared with the control (0.035 ± 0.043 μg vs. 0.120 ± 0.016 μg—biofilm; 3.332 ± 0.128 μg vs. 3.738 ± 0.152 μg—medium). As for the relative values after the first hour, the LpoS-I^−^ system exhibited a statistically significant ability to lower the pyruvate/biomass ratio in the biofilm. However, no significant differences were noted in the medium, unlike with the other systems, which significantly increased the ratio.

Three hours after exposure to various LpoSs, all of the tested systems caused a statistically significant reduction in pyruvate mass in both the biofilm and the medium, compared to the control. A significant reduction in the pyruvate/biomass ratio was observed in the samples where pyruvate was measured inside the biofilm after LpoS-I^−^ treatment. In the growth medium, the effect on the pyruvate/biomass ratio was similar to that observed after one hour—the iodide system did not significantly affect the ratio, while the other systems significantly increased it.

Five hours after exposure, the amount of the pyruvate inside the biofilms treated with LpoS-SCN^−^, LpoS-I^−^ and LpoS-SeCN^−^ remained at a statistically lower level. However, the decrease observed in LpoS-I^−^-SCN^−^ system was not statistically significant. At this measuring point, the amount of pyruvate in the growth medium was significantly lower only for the LpoS-I^−^ system. The relative pyruvate/biomass ratio values at the five-hour mark were significantly lower in both the biofilm and the growth medium for the LpoS-I^−^ system.

### 2.3. Assessment of the Metabolic Activity of Biofilms

**Figure 3:**
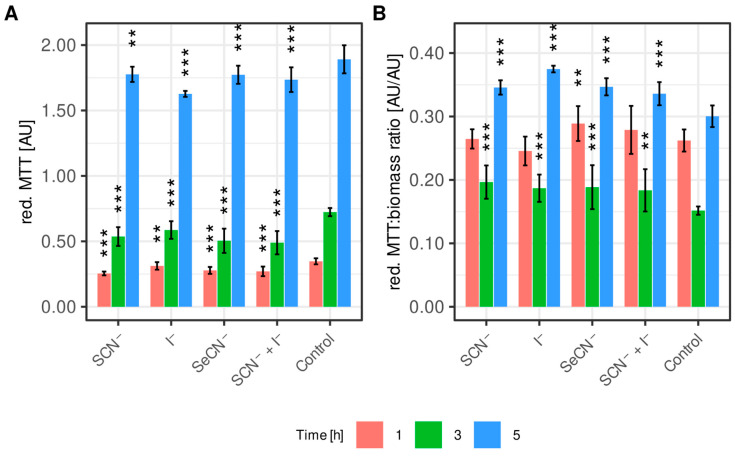
(**A**): Absorbance of MTT reduced by biofilms exposed to individual LpoS variants and the control sample. (**B**): Ratio of reduced MTT absorbance to the biomass of biofilms exposed to individual LpoS variants and the control sample. X-axis labels indicate the (pseudo)halide substrate used in each LpoS modification. Substrate controls (biofilms treated with (pseudo)halides or LPO alone) were omitted from the graphs for clarity—no effect of (pseudo)halides or LPO on the measured parameters was observed. Data are presented as mean ± SD. ** *p* < 0.01, *** *p* < 0.001 (Dunnett’s test).

Figure 3 shows the effect of the individual LpoSs on biofilm metabolism, expressed as the ability to reduce MTT to formazan (Figure 3A). When analyzing the direct measurement results expressed as the absorbance of the formed formazan, a statistically significant decrease in metabolic activity was observed in all cases (for all LpoSs modifications at all time points).

To correct for the influence of biomass growth inhibition caused by the individual systems, a statistical analysis was also performed after normalizing the absorbance of the formed formazan to the unit of biomass (Figure 3B). In this case, no significant effect of any LpoS on the analyzed parameter was observed after the first hour of incubation. However, after three and five hours, this parameter was significantly higher than in the control sample for each of the analyzed LpoSs.

### 2.4. The Effect of LpoSs on the Viability and Structure of the Biofilms

**Figure 4:**
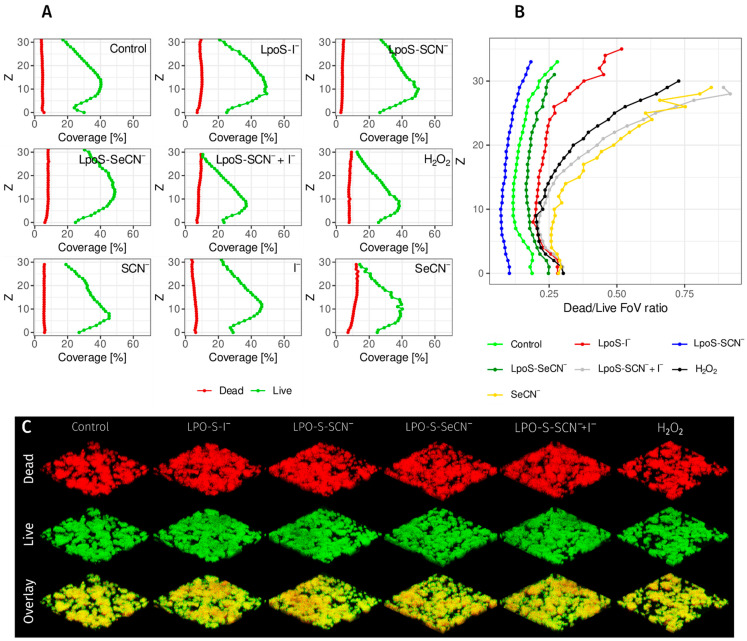
(**A**) The coverage of the field of view [%] with live and dead bacteria, depending on the Z-plane in CLSM. (**B**) The ratio of the field of view covered by dead bacteria to the field of view area covered by live bacteria on a specific plane. (**C**) Biofilm architecture observed by CLSM after SYTO9 staining (live cells—green) and propidium iodide (dead cells—red). The structures of the substrate controls were omitted from the illustrations to increase their clarity, as no differences were observed except in the sample treated with H_2_O_2_ and SeCN^−^, which are included in the illustration.

The structural analysis and viability of biofilms using the CLSM technique did not demonstrate a significant influence of the systems on parameters, except for the sample treated with LpoS-SCN^−^ + I^−^, where a reduction in the viability of the upper layers of the biofilm was observed. A notable decrease in the viability of the upper layers and coverage was observed in the substrate control containing H_2_O_2_ and the substrate control containing SeCN^−^. A significant decrease in the coverage was observed solely in the substrate sample containing H_2_O_2_ (Figure 4C).

## 3. Discussion

The study conducted demonstrates that each LpoSs modification has a different effect on the expression of virulence genes and pyruvate metabolism, while only having a marginal effect on the structure of the *S. mutans* biofilm. The manner in which the biofilm was treated with LpoSs during the experiments attempted to mirror the conditions in the oral cavity as closely as possible. The LPO concentration used in this study (50 nM, approximately 3.9 μg/mL) was selected to approximate physiological salivary levels, which range from 30 to 70 nmol/L [2]. This concentration is lower than that achieved in many commercial oral hygiene products. For example, Biotene^®^ toothpaste generates hypothiocyanite concentrations of 100–300 μM in vitro [21], and LPO-containing tablets deliver 1.8–2.6 mg per tablet [22]. However, despite using near-physiological enzyme concentrations, our experimental system generated approximately 250 μM of reactive products, which is comparable to the hypothiocyanite levels produced by commercial formulations. This was achieved through the use of optimized substrate concentrations (10 mM (pseudo)halide and 250 μM H_2_O_2_), demonstrating that the antimicrobial output depends not only on enzyme concentration but also on the availability of substrates. Studies by Lenander-Lumikari suggest that LPO activity should be 2–3 times higher than normal salivary levels (approximately 10 U/mL vs. 3–5 U/mL) for optimal antimicrobial effects [21]. Our findings, therefore, demonstrate that clinically relevant concentrations of reactive products can be achieved even at physiological enzyme levels when adequate substrate supply is ensured. Higher enzyme concentrations, as achievable through topical application of LPO-containing products, may produce even more pronounced effects on *S. mutans* biofilms [2]. The concentrations of each (pseudo)halide were set at 10 mM (for the SCN^−^ + I^−^ mixture, the sum equaled 10 mM), which was similar to the physiological concentration of the substrate (SCN^−^) in saliva [23]. The concentration of iodide in saliva remains at approximately ten times lower level than that of SCN^−^, and its presence does not significantly affect the physiological effects of salivary LpoS. The selenocyanate ion is completely absent from saliva. The last two substrates were included in the study as substances that can be supplied externally to modify the LPO system.

Some interesting results were obtained, particularly with regard to the expression of the *atpD* gene, which encodes the β-subunit of the F-ATPase and plays a pivotal role in the acid tolerance of *S. mutans*. The catalytic β-subunit of the F-ATPase proton pump, which is encoded by the *atpD* gene and is regulated in response to external pH, and its expression significantly increases in an acidic environment [19,24]. Significant decreases in *atpD* expression were observed in biofilms treated with LpoS-SCN^−^, LpoS-SeCN^−^ and LpoS-I^−^ + SCN^−^ systems, which suggests a possible mechanism by which the ability of bacteria to maintain pH homeostasis is impaired.

The optimal pH for *S. mutans* F-ATPase is around 6.0 (compared to pH 7.0 for *S. sanguis*), and it can function as both a proton pump in acidic conditions and an ATP synthase in conditions of low substrate availability [19,25]. This explains why *S. mutans* can dominate over other streptococci in the acidic environment characteristic of active dental caries.

The lowered expression of *atpD* caused by the tested LpoSs may have dual consequences. Firstly, it can directly impair the ability of bacteria to pump protons out of the cytoplasm, leading to the acidification of the cell interior and the distortion of metabolism. Secondly, it can limit ATP synthesis in stressful conditions, when bacteria use the proton gradient to produce energy. Research by Baker et al. shows that the loss of activity of other enzymes involved in restoring NAD+ leads to a compensatory increase in the expression of metabolic genes, including *ldh*, which is mediated by the redox sensor Rex [26]. In our case, however, the opposite situation can be observed: a simultaneous decrease in both *atpD* and *ldh* expression (in the case of the SCN^−^ and I^−^ systems), which may indicate a complex disruption to the bacterial adaptive response caused by individual LpoSs.

Hydrogen peroxide (250 μM) was used as the secondary LPO substrate at concentrations higher than physiological salivary levels, which are difficult to measure due to rapid turnover between production and consumption. Since H_2_O_2_ was added at lower molar quantities than (pseudo)halides, it served as the limiting substrate determining the yield of reactive products. For SCN^−^ and SeCN^−^ systems, the generated OSCN^−^/HOSCN was stoichiometrically equivalent to the added H_2_O_2_, whereas LpoS-I^−^ and LpoS-SCN^−^ + I^−^ produced mixtures of reactive species that may undergo secondary reactions. After analysis, it was shown that the (pseudo)halide substrate controls did not affect the tested parameters except for SeCN^−^, which affected gene expression (*ldh*, *gftB*), as well as viability and structure of the biofilm. The influence of the substrate in question may be connected to the phenomenon of spontaneous decomposition of used KSeCN to atomic selenium and highly toxic cyanide ions (CN^−^) in the presence of oxygen. Despite the high concentration of H_2_O_2_ and toxic effect in substrate control (i.e., biofilm treated with H_2_O_2_) that was observed, the effects of biofilm treatment with LpoS should be linked to the impact of the generated products of LpoS, rather than the toxic effect of H_2_O_2_. This phenomenon can be attributed to the addition of H_2_O_2_ to the mixture in the final stage of the procedure, a step that immediately triggered the enzymatic reaction. Based on the kinetic properties of the LPO system at the used substrate concentrations and consistent with previous reports [27], and based on our own observations (Section 4.2), the consumption of all available H_2_O_2_ in the mixture was estimated to occur within seconds. This brief exposure time, as evidenced by other studies, is insufficient to elicit a toxic effect.

The synthesis of lactic acid is a key factor in the carcinogenicity of *S. mutans* biofilms. As demonstrated in the preceding study, the LpoS-I^−^ system was found to possess the capacity to entirely halt the production of lactic acid in biofilm cultures, in addition to reducing the NADH content within the biofilm [1]. The present study was conducted with the objective of investigating the mechanism of action of the system in question, with a focus on establishing whether the observed effect was associated with alterations in gene expression or direct influence on enzymes and metabolites. The present study determined that in biofilms treated with LpoS-I^−^ a decline in pyruvate synthesis and a decrease in *ldh* gene expression were observed. Nevertheless, the latter was not significant enough to inhibit lactic acid synthesis due to insufficient enzyme expression. Taking this into consideration, it can be concluded that the effect of this system on biofilms is multidirectional and involves influencing *lhd* expression, as well as the oxidation and decomposition of NADH (a substrate for LDH), and the influence on other glycolysis reactions, which cause a reduction in pyruvate synthesis.

Ahn et al. demonstrated that *S. mutans* in logarithmic growth expels excess pyruvate to maintain intracellular redox balance and prevent acidification, with subsequent re-uptake via the LrgAB system after carbohydrate depletion [28]. Our study observed similar intense pyruvate excretion in both control and treated samples. Notably, all systems except LpoS-I^−^ showed significantly elevated pyruvate/biomass ratios in the medium at 1 and 3 h, without affecting intracellular ratios. Since pyruvate export serves as a protective mechanism against oxidative stress [29], the unchanged extracellular Pyr:BM ratio after LpoS-I^−^ treatment may indicate impairment of this protective response. The precise impact on pyruvate transporters remains difficult to assess due to limited understanding of the export mechanism in the literature. [28,29]. “A methodological consideration of this study is that crystal violet staining, used for biomass normalization of pyruvate and MTT data, binds to both bacterial cells and extracellular matrix components, including polysaccharides, proteins, and extracellular DNA [30,31]. This is a recognized inherent limitation of CV-based biomass quantification in biofilm research, as treatments that differentially affect matrix production could potentially influence normalized values. In *S. mutans* biofilms, where glucans synthesized by glucosyltransferases constitute a major matrix component, this consideration is particularly relevant. To address this limitation, we employed a complementary methodological approach. First, the expression of the *gtfB* gene, encoding glucosyltransferase B responsible for water-insoluble glucan synthesis, was analyzed by qRT-PCR, providing direct information about the matrix production capacity of treated biofilms. Second, confocal laser scanning microscopy (CLSM) with LIVE/DEAD staining was performed, which enables direct visualization and quantification of bacterial cells independent of matrix components. The SYTO9/propidium iodide staining specifically targets bacterial DNA, allowing assessment of cell viability without interference from the extracellular matrix. The consistency between CLSM-derived structural and viability data and the CV-normalized metabolic findings supports the validity of our conclusions and indicates that the observed effects were not artifacts of matrix-related normalization bias. This multi-method approach, combining biochemical assays with microscopic analysis and gene expression profiling, provides a more comprehensive and reliable assessment of LPO system effects on *S. mutans* biofilms than any single method alone.”

The analysis of *ldh* expression, which encodes lactate dehydrogenase, revealed a multifaceted picture of bacterial response to the action of different LpoS modifications. *S. mutans* LDH is an enzyme that catalyzes a reversible conversion of pyruvate to lactate, with simultaneous regeneration of NAD^+^ from NADH [32]. Deletion of *ldh* is lethal for *S. mutans* under in vitro conditions, thereby emphasizing the critical role it plays in the survival of the bacteria [33].

The study demonstrated that both LpoS-SCN^−^ and LpoS-I^−^ exhibited a significant decrease in *ldh* expression, while LpoS-SeCN^−^ demonstrated an increase in this parameter. The regulation of LDH activity is dependent upon the intracellular level of fructose-1,6-diphosphate (FDP). In conditions of excess glucose, the FDP level is elevated, which activates LDH and leads to lactate production. Conversely, when glucose is in low supply, the low FDP level results in transition to mixed fermentation [34]. Lowering *ldh* expression by the SCN^−^ and I^−^ systems may not only directly limit lactic acid production, but also disrupt the bacteria’s metabolic adaptability in response to a changing environment. The increased *ldh* expression in response to LpoS-SeCN^−^ may be connected to the influence of the substrate (SeCN^−^) itself, as the same effect was observed in the substrate control sample.

Gaspar et al. demonstrated that the absence of LDH in *Streptococcus pneumoniae* results in the accumulation of pyruvate and a shift in metabolism towards ethanol production by alcohol dehydrogenase. This process contributes to maintaining redox equilibrium [35]. The study revealed a decline in both the intracellular and extracellular concentrations of pyruvate following LpoS-I^−^ treatment. This finding indicates a disruption of glycolysis that is more intricate than simply an obstruction at the stage of pyruvate conversion.

Unexpectedly, LpoS exposure did not decrease metabolic activity measured by MTT reduction; instead, an increase was observed at 3 and 5 h post-exposure. Although absolute MTT values were statistically lower in treated samples, this difference was biologically marginal, and when normalized to biomass, it reflected reduced biomass growth rather than direct metabolic inhibition. This effect may represent a post-stress metabolic rebound. Reactive LPO products are unstable and act within minutes of formation, rapidly reacting with target chemical groups before decomposing. Consequently, cellular damage occurs immediately after exposure, and the inability of bacteria to repair their metabolism—as observed at 3 and 5 h timepoints—would result in inhibited biomass growth and eventual cell death. The analysis of biofilm microstructure with CLSM confirmed that under the conditions of the experiment, LpoS did not have a bactericidal effect on *S. mutans*. The viability of *S. mutans* in biofilms was not statistically significantly different, with the exception of the samples containing LpoS-SeCN^−^, where, similarly to the experiments described above, the observed effect can be attributed to the influence of selenocyanate itself, as the decrease in the viability was also observed in the substrate control. Moreover, a marked decline in viability was evident in the upper layers of each sample in comparison to the lower layers. This phenomenon has also been documented by other research teams [36,37]. The phenomenon may be associated with the experimental procedure. Specifically, biofilms are deprived of the growth medium before treatment with systems and during staining and repeatedly rinsed, which exposes them to sudden changes in conditions. The internal layers of biofilm are protected by the external layers, thereby ensuring that alterations within the biofilm occur at a slow and gradual pace, dependent on diffusion through the biofilm. It can thus be concluded that the observed and published decrease in biofilm biomass subsequent to the addition of LpoS is not a consequence of a decrease in the quantity of microorganisms, but rather a consequence of a decrease in the quantity of extracellular matrix. It was confirmed in the assay of the *gftB* gene expression responsible for production of glucosyltransferase, an extracellular enzyme that synthesizes glucan, the main element of extracellular matrix of the *S. mutans* biofilm. With the exception of LpoS-I^−^, each system demonstrated the capacity to reduce *gftB* expression. This outcome is not immediately apparent, particularly when considering that this system exhibited the most pronounced inhibition of biofilm biomass growth. The findings of both studies suggest that the impact of individual LpoS on *S. mutans*’ capacity to synthesize biomass is not a unidirectional effect, but rather a multidirectional effect on gene expression and the function of proteins involved in matrix development.

Several limitations of this study should be acknowledged when considering the clinical translation of these findings. The efficacy of the modified LPO systems was not compared with established oral antimicrobial agents such as chlorhexidine, cetylpyridinium chloride, or fluoride-based formulations, which would provide valuable clinical benchmarking. However, direct comparison may be complicated by fundamentally different mechanisms of action—conventional antiseptics act primarily through membrane disruption or metabolic poisoning, while LPO-derived products exert their effects through selective thiol oxidation and metabolic interference. Future studies should include such comparative analyses to better contextualize the clinical potential of modified LPO systems within the spectrum of available anticaries agents. Furthermore, this study utilized a single-species *S. mutans* biofilm model, which, while allowing precise mechanistic investigation, does not fully reflect the polymicrobial complexity of dental plaque in vivo. The oral cavity harbors a diverse microbial community where interspecies interactions significantly influence biofilm behavior, metabolic activity, and susceptibility to antimicrobial treatments. Future research should therefore validate these findings using multispecies biofilm models that better simulate the clinical environment, as well as in situ and clinical studies to confirm the therapeutic potential of iodide-modified LPO systems in caries prevention.

To summarize, the present study demonstrates a complex and multilevel mechanism of action of modified lactoperoxidase systems on *S. mutans* biofilms. The most promising system was found to be LpoS-I^−^, which resulted in a reduction in the expression of key virulence genes (*atpD* and *ldh*), an inhibition of the synthesis and export of pyruvate, and a decrease in lactate production. The mechanism of action of this system consists of both modulation of gene expression and the effect on enzymes and metabolites of the glycolytic pathway. Especially important is the simultaneous impairment of two main mechanisms of pathogenicity in *S. mutans*, the acid tolerance, which is achieved by decreasing the expression of *atpD* that encodes the F-ATPase, and decreasing lactic acid production by *ldh* suppression. Despite the fact that the investigated systems did not demonstrate direct bactericidal action, their capacity to impair the key metabolic processes and disrupt the structure of biofilms renders them a potentially useful instrument in the prevention of dental caries. The observed effect of metabolic rebound after exposure to LpoS systems indicates the necessity for the development of a strategy of extended or repeated exposure to prevent the adaptation of bacteria to oxidative stress. Further studies should concentrate on the optimization of LpoS-I^−^ system operating conditions and on investigating its practical application in oral hygiene products.

## 4. Materials and Methods

### 4.1. Biofilm Culture Conditions

In the present study, *S. mutans* ATCC 25175 strain was used. It was grown using multi-well plates in Brain Heart Infusion medium (BHI) (Graso Biotech, Owidz, Poland) supplemented with 5% sucrose at 37 °C and atmosphere supplemented with 10% CO_2_. The duration of biofilm growth, the initial suspension density and type of multi-well plates varied depending on the type of experiment. The exact conditions for growth can be found in the relevant subchapters.

### 4.2. LPO System

The LPO concentration was 50 nmol/L in each experiment. Lyophilized enzyme (Sigma-Aldrich, Saint Louis, MO, USA) was dissolved in PBS, and the absorbance at 412 nm was measured to determine the enzyme concentration (ε_412_ = 112,000). Enzyme activity was verified before each experiment by a rapid spectrophotometric assay. In a 96-well plate, 200 μL of PBS containing hydrogen peroxide (250 μM), potassium iodide (10 mM), and LPO (50 nM) were combined, and absorbance at 400 nm was monitored. Completion of the reaction within 10 s and final absorbance exceeding 0.550 confirmed enzyme activity consistent with manufacturer’s specifications (≥200 units/mg protein). (Pseudo)halide substrates (KSCN, KI and KSeCN) (Chempur, Piekary Śląskie, Poland) were added to the reaction mixture at a concentration of 10 mmol/L. Hydrogen peroxide was always added last to initiate the enzymatic reaction, achieving a final concentration of 250 μM. Incubation with the LPO system lasted 15 min in each experiment and was followed by discarding the supernatant fluid, rinsing the biofilm with PBS, and adding fresh BHI + 5% sucrose medium to resume biofilm metabolism under standardized conditions before continuing with the subsequent procedures as described. The control sample in all of the experiments consisted of PBS without the enzyme or substrates.

### 4.3. CV Biofilm Biomass Assay

The biofilm was grown in 96-well plates. Once the incubation period had finished, the biofilms were rinsed three times. Then, 100 μL of 0.1% crystal violet solution (Chempur, Piekary Śląskie, Poland)was added to each well, and the plate was incubated at 37 °C for 20 min to allow the dye to bind to the structure. The dye was then discarded, and the biofilms were rinsed with PBS until all unbound CV had been removed. The bound dye was extracted with a 3% acetic acid solution (Chempur, Piekary Śląskie, Poland), and the absorbance of the solution was then measured on a fresh plate; this measurement was proportional to the total biomass of the biofilm [38].

### 4.4. Pyruvate Assay

A pyruvate concentration assay was conducted in both the supernatant medium (pyruvate expelled from the cells) and inside the bacterial cells. The experiment was carried out on a 2 h biofilm grown in 24-well plates. To achieve this, 1 mL of a 0.1 McF *S. mutans* suspension was added to each well, after which the plates were incubated for 2 h under the conditions described above. They were then treated with the LPO system. The medium was then changed to a fresh portion of BHI + S, and the plates were incubated until the time of the measurement. Measurements were taken at three time points: after 1, 3, and 5 h following treatment of the biofilm with the LPO system. The pyruvate concentration was measured in the supernatant medium removed directly from the biofilm culture. To assess the intracellular pyruvate concentration, the biofilm was rinsed three times with PBS and once with pure water after the incubation period. The supernatant was then discarded, and the plate was frozen at −20 °C before being lyophilized to expel any remaining water. The wells containing the dehydrated biofilm were then filled with 150 μL of 50% ethanol, the biofilm was scraped off and homogenized by repeated aspiration and dispensing using a pipette tip. The resulting suspension was transferred to an Eppendorf tube. To recover all of the pyruvate, the well was rinsed twice with 100 μL of 50% ethanol. The suspension was incubated at room temperature for 30 min, with shaking every 5 min to extract the intracellular pyruvate. Finally, it was centrifuged for 5 min at 550 RCF, and the supernatant was acquired for further analyses. The extraction protocol using 50% ethanol was selected following optimization experiments comparing four extraction reagents (0.5 M HCl, 0.1% SDS, 5% TCA, and 50% ethanol) for their efficiency in releasing pyruvate from bacterial biofilms while maintaining compatibility with the enzymatic assay.

Pyruvate concentration was measured using a colorimetric method in the supernatant and a fluorimetric method in cell extract, both of which utilized pyruvate oxidase in the ready-to-use Pyruvate Assay Kit MAK332-1KT (Sigma-Aldrich, Saint Louis, MO, USA). The results were then recalculated to determine the pyruvate mass content in one well (supernatant) or in one biofilm (intracellular pyruvate). For the statistical analysis, the result was divided by the total biofilm biomass in the parallel sample (measurement was taken under identical conditions) to compensate for the effect of biomass-related pyruvate production.

### 4.5. MTT Assay

The procedure began with the preparation of the staining solution by mixing 5 mL of MTT solution (5 mg/mL), 10 mL of BHI medium supplemented with 5% sucrose, and 35 mL of PBS. To determine the metabolic activity of the cells, each well of a 96-well plate (2 h biofilm) was filled with 150 µL of the prepared solution, and the plates were then incubated for 30 min at 37 °C in an atmosphere containing 10% CO_2_.

After incubation, the supernatant was carefully removed. Subsequently, 180 µL of DMSO was added to each well to dissolve the formazan crystals. The plates were gently shaken for 15 min at room temperature. After this period, the contents of each well were transferred to a clean plate, and absorbance was measured at a wavelength of 570 nm using a Thermo Scientific Multiskan Sky spectrophotometer (Thermo Fisher Scientifi, Waltham, MA, USA) [39].

### 4.6. RNA Isolation

Total RNA from the biofilm was isolated using a modified phenol–chloroform extraction method based on the procedure described by Cury and Koo [40]. The 2 h biofilms were rinsed three times with 1 mL of PBS to remove residual growth medium. The biofilm was then scraped from the bottom of each well using a 200 µL pipette tip, and 900 µL of PBS was added. The contents were repeatedly aspirated and dispensed to obtain a homogeneous suspension, which was transferred to a fresh Eppendorf tube. Each well was subsequently rinsed twice with 250 µL of PBS to recover all remaining material.

The samples were centrifuged at 5500× *g* for 10 min at 4 °C. The supernatant was discarded, and the pellet was resuspended in 400 µL of 50 mM acetate buffer (pH 5.0) containing 10 mM EDTA and 1% SDS. Next, 400 µL of RNAzol RT (Sigma-Aldrich, St. Louis, MO, USA), approximately 150 µL of acid-washed glass beads (<106 µm; Sigma-Aldrich), and 80 µL of chloroform were added. The tubes were vortexed for 30 s and subjected to ultrasonic homogenization (Hielscher UP100H) (Hielscher Ultrasonics, Teltov, Germany) for 15 s. The samples were then incubated at room temperature for 5 min, with vortexing for 15 s every minute.

After incubation, the samples were cooled on ice and centrifuged at 10,000× *g* for 5 min at 4 °C, and the aqueous phase was collected into chilled Eppendorf tubes. The remaining material was extracted twice more by adding 400 µL of RNAzol and 80 µL of chloroform, followed by vortexing and centrifugation under the same conditions. An equal volume of an isoamyl alcohol–chloroform mixture (24:1) was added to the pooled aqueous phase, vortexed, and centrifuged at 14,000× *g* for 5 min at 4 °C. The aqueous phase was collected, and 1/10 volume of 3 M sodium acetate (pH 5.0) and an equal volume of ice-cold isopropanol were added. The samples were frozen at −20 °C to precipitate RNA, then centrifuged at 14,000× *g* for 15 min at 4 °C. The supernatant was discarded, and the pellet was washed twice with isopropanol under the same conditions. The pellet was then resuspended in nuclease-free, molecular biology-grade water. RNA concentration was determined with a NanoDrop 2000 spectrophotometer (Thermo Fisher Scientific, Waltham, MA, USA). Excess DNA was removed from the sample with DNase I (Thermo Fisher Scientific, Waltham, MA, USA), according to the manufacturer’s recommendations, using up to 1 U of DNase I per 1 µg of RNA.

RNA purity and concentration were assessed spectrophotometrically by determining the A260/A280 ratio using NanoDrop 2000 (Thermo Fisher Scientific, Waltham, MA, USA). Samples with ratios above 1.7 were selected for further analysis.

### 4.7. qRT-PCR Analysis

Total RNA was reverse-transcribed into cDNA using the Applied Biosystems High-Capacity cDNA Reverse Transcription Kit (Thermo Fisher, Scientific Waltham, MA, USA). Primers were commercially obtained from Genomed S.A. (Warsaw, Poland), and their sequences were selected based on Wu et al. [41] (Table 1).

Quantitative real-time PCR (qRT-PCR) assays were performed on a QuantStudio 12K Flex Real-Time PCR System (Thermo Fisher) under the following reaction conditions: 95 °C for 10 min, 40 cycles per 95 °C for 15 s and 60 °C for 30 s, followed by a melting curve analysis. The expression levels of the *16S rRNA*, *gyrA*, *gtfB*, *atpD* and *ldh* genes were measured. Relative gene expression was calculated using the 2^(−ΔΔCT)^ method.

### 4.8. Confocal Laser Scanning Microscopy

The three-dimensional structure depicting the distribution of living and dead microorganisms was observed using a Zeiss LSM780 CLSM (Carl Zeiss, Jena, Germany) Biofilms were cultivated in an 8-chamber Lab-Tek system (Nunc, Rochester, NY, USA). For this purpose, 200 μL of a 0.1 McF suspension of *S. mutans* was added to each chamber, and the biofilms were incubated for 2 h. The supernatant was then collected, and the biofilms were rinsed three times with PBS, and then the biofilms were subjected to LPO systems. After collecting the supernatant, the biofilms were rinsed three times with 0.9% NaCl. Live/dead staining was performed using a LIVE/DEAD™ BacLight™ Bacterial Viability Kit (Thermo Fisher, Waltham, MA, USA). Ready staining solution contained 8.35 μM and 50 μM of SYTO9 and propidium iodide, respectively. The biofilms were incubated with the staining solution for 20 min at room temperature. After discarding the supernatant, the sample underwent CLSM analysis. The functionality of the SYTO9/PI staining was confirmed by the H_2_O_2_ substrate control, which showed the expected increase in PI-positive (dead) cells, consistent with the known bactericidal effect of hydrogen peroxide. Observations were performed using a 20× objective lens. Photographs were taken of representative areas of the biofilms as a Z-stack, covering the full thickness of the sample. Viability analysis of the cross-section was performed using an original Python (version 3.13) script written by the authors and posted on the GitHub platform.

### 4.9. Statistical Analyses

The data obtained were analyzed using the R Environment, version 4.5.1. The significance level for all statistical tests was set at α = 0.05. Data normality was assessed using the Shapiro–Wilk test and homogeneity of variance was evaluated using Levene’s test. ANOVA was used to compare statistically significant differences between multiple groups, followed by a post hoc Dunnett’s test.

## 5. Conclusions

The present study demonstrates that modified lactoperoxidase systems exert distinct effects on *S. mutans* biofilms, with each (pseudo)halide substrate producing a unique pattern of metabolic and transcriptional modulation. Among the tested variants, LpoS-I^−^ emerged as the most promising system, effectively targeting multiple virulence mechanisms simultaneously. This system reduced the expression of *atpD* and *ldh* genes, impairing both acid tolerance and lactic acid production—two critical factors in the cariogenic potential of *S. mutans.* Additionally, LpoS-I^−^ was the only variant that significantly decreased both intracellular and extracellular pyruvate levels, indicating a comprehensive disruption of glycolytic flux rather than inhibition at a single enzymatic step. The LpoS-SCN^−^ and LpoS-SeCN^−^ systems demonstrated significant downregulation of *atpD* and *gtfB* expression, suggesting potential effects on acid tolerance and extracellular matrix synthesis. However, these systems showed less pronounced effects on pyruvate metabolism compared to LpoS-I^−^. Notably, despite using near-physiological enzyme concentrations, the experimental system generated clinically relevant levels of reactive products comparable to commercial oral hygiene formulations. Although no direct bactericidal action was observed, as confirmed by CLSM analysis, the capacity of modified LpoS to comprehensively impair bacterial metabolism and inhibit biofilm biomass growth supports their potential as active ingredients in anticaries products. Future studies should focus on validating these findings in multispecies biofilm models, optimizing formulation parameters, and conducting clinical trials to confirm the therapeutic efficacy of iodide-modified lactoperoxidase systems in caries prevention.

## Figures and Tables

**Table 1:** Primers sequence used for quantitative real-time PCR.

Gene	Primers	Tm (°C)	Product Size (bp)
*16S rRNA*	F: ACCAGAAAGGGACGGCTAAC R: TAGCCTTTTACTCCAGACTTTCCTG	59.68 60.28	123
*gyrA*	F: ATTGTTGCTCGGGCTCTTCCAG R: ATGCGGCTTGTCAGGAGTAACC	63.15 62.89	105
*atpD*	F: GGCGACAAGTCTCAAAGAATTG R: AACCATCAGTTGACTCCATAGC	58.19 58.12	91
*gtfB*	F: AGCAATGCAGCCATCTACAAAT R: ACGAACTTTGCCGTTATTGTCA	58.97 59.13	96
*ldh*	F: ACTTCACTTGATACTGCTCGTT R: AACACCAGCTACATTGGCATGA	57.74 60.56	141

## Data Availability

The original contributions presented in this study are included in the article/Supplementary Materials. Further inquiries can be directed to the corresponding authors.

## Supplementary Materials

The following supporting information can be downloaded at https://github.com/mmagacz/clsmviability/tree/main (accessed on 20 December 2025).

## Abbreviations

The following abbreviations are used in this manuscript:

BHI	Brain heart infusion medium
CLSM	Confocal laser scanning microscope
CV	Crystal violet
DMSO	Dimethyl sulfoxide
LDH	Lactate dehydrogenase
LpoS	Lactoperoxidase system
MTT	3-(4,5-dimethylthiazol-2-yl)-2,5-diphenyltetrazolium bromide
PBS	Phosphate-buffered saline
RCF	Relative centrifugal force
